# Preliminary Histological Evaluation of the Application of Ozone in the First Days of Orthodontic Force Induction in Animal Model

**DOI:** 10.1055/s-0041-1731886

**Published:** 2021-08-24

**Authors:** Melissa Faccini, Felipe Agostini, Tassio Drieu, Francisco Ubiratan Ferreira de Campos, Aguinaldo Garcez, Glauber Fabre Carinhena, Samira Salmeron, Ana Regina Casaroto, Fabricio Pinelli Valarelli, Karina Maria Salvatore Freitas

**Affiliations:** 1 Department of Orthodontics, Ingá University Center UNINGA, Maringá, Brazil; 2 Department of Dentistry, São Leopoldo Mandic, Campinas, Brazil; 3 Department of Oral Microbiology, São Leopoldo Mandic, Campinas, Brazil; 4 Department of Orthodontics, Superior Dentistry School, Brazil; 5 Department of Periodontics, Ingá University Center UNINGA, Maringá, Brazil; 6 Department of Pathology, Ingá University Center UNINGA, Maringá, Brazil; 7 Department of Orthodontics, Ingá University Center UNINGA, Maringá, Brazil

**Keywords:** orthodontic movement, ozone, orthodontics, ozone therapy

## Abstract

**Objectives** The aim of the study was to histologically evaluate the effect of ozone therapy on orthodontic force induction in an animal model.

**Materials and Methods** Twenty-four Wistar rats were divided into three groups ( *n* = 8). A NiTi coil spring was installed from the maxillary first molar to the maxillary central incisor. G1 was control and G2/G3 received 1 mL of ozonated gas at concentrations of 10 and 60 µg/mL, in the buccal mucosa above the first molar roots. The animals were euthanized 3 and 5 days after the procedure. Histological sections were obtained, longitudinally of the first molar’ long axis, in the mesiodistal direction. The number of osteoclasts, osteoblasts, blood vessels, polymorphonuclear and mononuclear cells, formation of osteoid tissue and hyaline areas, and root resorption were evaluated with light microscope, in tension and pressure sides. Intergroup comparisons were performed with Kruskal–Wallis, Dunn, and Chi-square tests.

**Results** At 3-days pressure side, a greater number of osteoclasts was observed in ozone groups and greater number of blood vessels and polymorphonuclear cells were observed in G2. On the tension side, there was a significantly greater number of blood vessels, osteoblasts, and mononuclear cells in G2. At 5-days pressure side, there was a significantly greater number of osteoclasts in G2, blood vessels and osteoblasts in the ozone groups, and lesser number of polymorphonuclear cells in G3.

**Conclusion** Ozone therapy increased the number of osteoclasts on the pressure side and osteoblasts on tension side, in 10 µg/mL concentration, demonstrating histological parameters favorable to bone remodeling. The 60 µg/mL ozone concentration accelerated the periodontal ligament reorganization process.

## Introduction

Modern orthodontics is always seeking techniques that aim to accelerate orthodontic treatment. Surgical techniques, such as corticotomies 1 and minimally invasive therapies, such as piezocision 2 and bone microperforations, 3 promise the acceleration of orthodontic movement. Also mentioned are nonsurgical methods such as low-level laser therapy, 4 5 and extra or intraoral vibration devices. 6 All of these therapies promise to recruit cells that are important for efficient tooth movement. New techniques are still being tested, such as platelet-rich plasma. 7 However, none of them presents high level of scientific evidence of accelerating orthodontic tooth movement. 8 Low-quality evidence indicates that low-level laser therapy and corticotomy are effective to accelerate tooth movement in the short term. 5 8

Searching for a less invasive alternative to accelerate orthodontic treatment, the use of ozone was considered. Ozone therapy is used in several countries as a complementary health care treatment, including dental procedures. Ozone is obtained in dental offices through an ozone generator that, from medical oxygen and electrical discharges, generates ozone molecules. It is a treatment of easy acceptance and quick application, viable as a complementary therapy. 9 Its benefits (bactericidal, immunostimulant, anti-inflammatory, healing) are already proven and used in endodontics, restorative dentistry, periodontics, and oral surgery. 9 10 11 12 13

In orthodontics, only one study evaluated the action of the ozonated gas after maxillary expansion in rats and found increased bone regeneration during the retention period, in a concentration of 25 µg/mL. 14 Some orthodontic studies have also evaluated the bactericidal action of ozone through the use of ozonated water. 15 16 17 The use of ozonated water was also evaluated prior to bracket bonding with the purpose of improving the adhesive strength. 18 19 20 There is no known study evaluating the effects of ozone therapy on orthodontic force induction.

The ozone is biostimulator, recruiting important cells of the immune system through a complex biochemical reaction of redox. 21 Thus, ozone therapy can act to modulate inflammatory response, improving repair processes, 14 stimulating or even suppressing the immune system. 22

Medicinal ozone therapy has also been shown to increase the number of inflammatory mediators such as interleukins [Il] 1 β, IL-6, IL-8, tumor necrosis factor α, and cytokines, 23 24 which are important for tooth movement. In addition, ozone also has analgesic action, 22 25 promoting excellent microcirculation 14 21 and increasing the number of osteoblasts and osteoclasts when used in low concentration. 14 Ozone therapy has been used for bone remodeling of herniated disks 26 ; there are reports of analgesia promoted by modulation of inflammation 22 and stimulation of bone regeneration in intermaxillary sutures, 14 advantages that can be explored in Orthodontics. However, there is still no known study evaluating the effects of ozone therapy in orthodontically-induced tooth movement.

This way, the present study aimed to histologically evaluate the effects of ozone therapy during the first days of orthodontically-induced tooth movement in an animal model.

## Material and Methods

This study was approved by the Animal Research Ethics Committee (IRB 2018/043) following the guidelines of ARRIVE (Animal Research: Reporting of In Vivo Experiment).

The sample size calculation was performed on G*Power 3.1.5 software, adopting the model of analysis of variance. For the effect size of 0.84, obtained from the means and standard deviations presented in a previous study, 14 significance level of 5%, power of 80%, and loss factor of 0.2, the results indicated the need of eight animals in each group, four animals in each subgroup.

Twenty-four male *Rattus norvegicus* —Wistar, weighing around 300 g each and approximately 3 months of life were kept in a controlled environment with standard 12-hour light/dark cycle, under constant temperature of 23°C. Animals were fed with crushed feed and water *ad libitum* .

The animals were randomly divided into three groups according to the application or not of ozonate gas in different concentrations ( *n* = 8).

Group 1 (control) received only the coil spring for induced tooth movement and no ozone therapy. Group 2 received the coil spring plus application of ozonated gas with concentration of 10 µg/mL. Group 3 received the coil spring and application of ozonated gas with concentration of 60 µg/mL. In all groups, half of the animals ( *n* = 4) were euthanized 3 days after the procedure and the other half after 5 days.

For the placement of the orthodontic device, the rats were anesthetized with a mixture of anesthetic and muscle relaxant in the appropriate dosage (12 mg/kg of xylazine hydrochloride and 90 mg/kg of ketamine hydrochloride) applied intraperitoneally with 1 mL syringe. The rats were placed on a stretcher that provided the mouth opening through two fixed ends and placement of intermaxillary elastics.

For the orthodontic force induction, the mesial inclination of the right maxillary first molar was required, using a device adapted from Heller and Nanda. 27 The orthodontic device consisted of a 7-mm NITI closed coil spring (Orthometric, Marília, Brazil) tied by 0.020-mm stainless steel ligature wire at the extremities (Morelli, Sorocaba, Brazil).

**Fig. 1:**
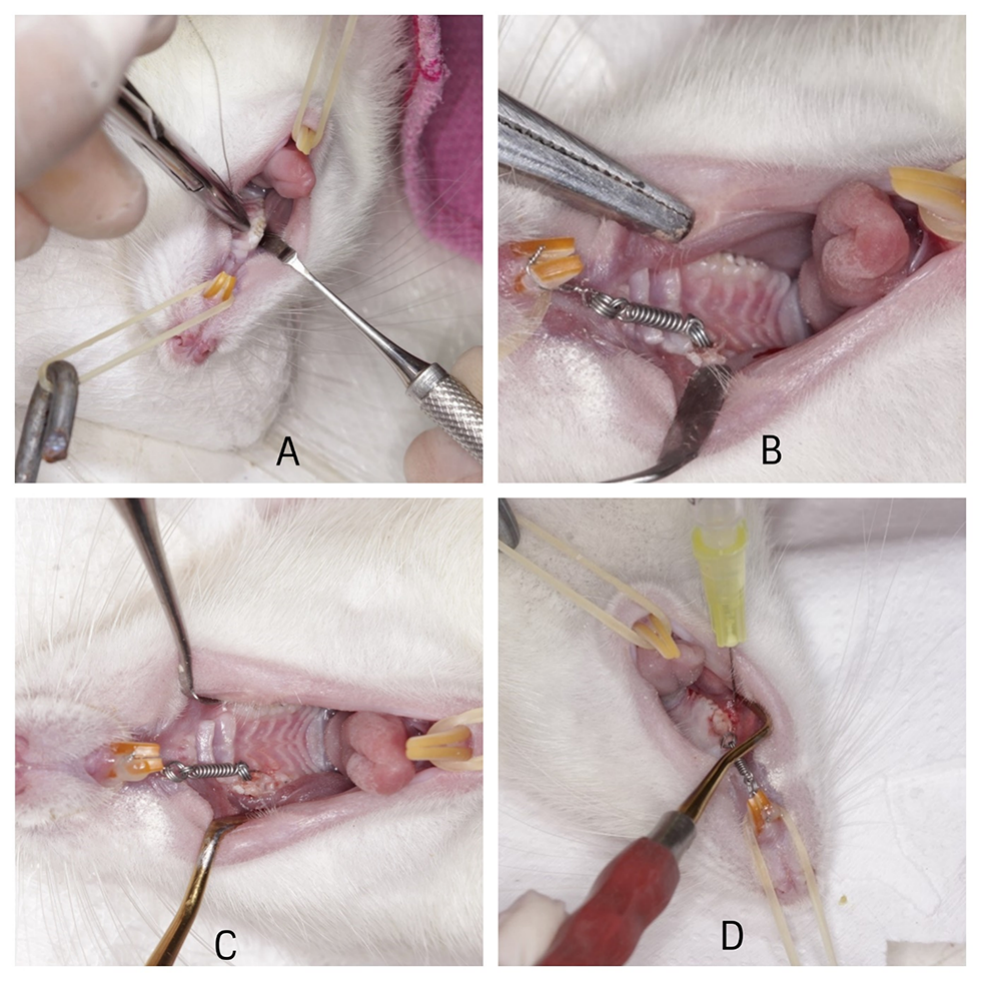
Installation of the orthodontic device and ozone gas application. ( **A** ) Insertion of the steel ligature wire between the first and second molars; ( **B** ) Device installed with the coils spring fixed in both incisor and first molar; ( **C** ) Photoactivated composite resin added to the incisor; ( **D** ) Application of the ozonated gas.

In the posterior region, the ligature wire was inserted between the maxillary first and second molars, contouring the cervical of the crown of the first molar ( Fig. 1A ). In the anterior region, the ligature wire was fixed around the right maxillary central incisor, in a groove created with carborundum disk close to the gingival margin ( Fig. 1B ). Photoactivated composite resin was added to fix the ligature wire to the incisor ( Fig. 1C ). A force of 50 g was applied, measured with a precision tensiometer. There was no force reactivation during the experimental period.

Ozonated gas in concentrations of 10 and 60 µg/mL was obtained from an ozone generator (Philozon, Balneário Camboriú, Brazil). This device features self-calibration, with automatic stabilization. The medical oxygen used (99.5%, White Martins, Rio de Janeiro, Brazil) has a regulated flow rate of 1 L/min. The ozone generator dispensed the gas directly in a 1-mL sterile silicone syringe (Solidor, Osasco, Brazil). A 0.30 × 13 mm disposable needle was attached to the syringe (BD, Curitiba, Brazil).

In groups 2 and 3, 1 mL of ozonated gas was applied to the buccal mucosa above the maxillary first molar roots ( Fig. 1D ). Only the needle bevel was inserted. The ozone therapy was performed only once, right after installation of the coil spring.

After 3 and 5 days, the animals were euthanized with an overdose of anesthesia (lidocaine 10 mg/mL, and after 10 minutes, intraperitoneal application of sodium thiopental 150 mg/kg).

Samples collected were fixed in 10% buffered formaldehyde for 24 hours and decalcified in a 20% formic acid solution (Merck, Darmstadt, Germany) for 7 days. Then, the pieces were dehydrated, diaphanized, and embedded in paraffin, and histological cuts of 4 μm thickness were made. The sections were made in the mesiodistal direction in the maxillary first molar, parallel to the long axis showing the mesiobuccal and distobuccal roots. Subsequently, the material was stained with hematoxylin and eosin (HE) for qualitative and quantitative analyses.

**Fig. 2:**
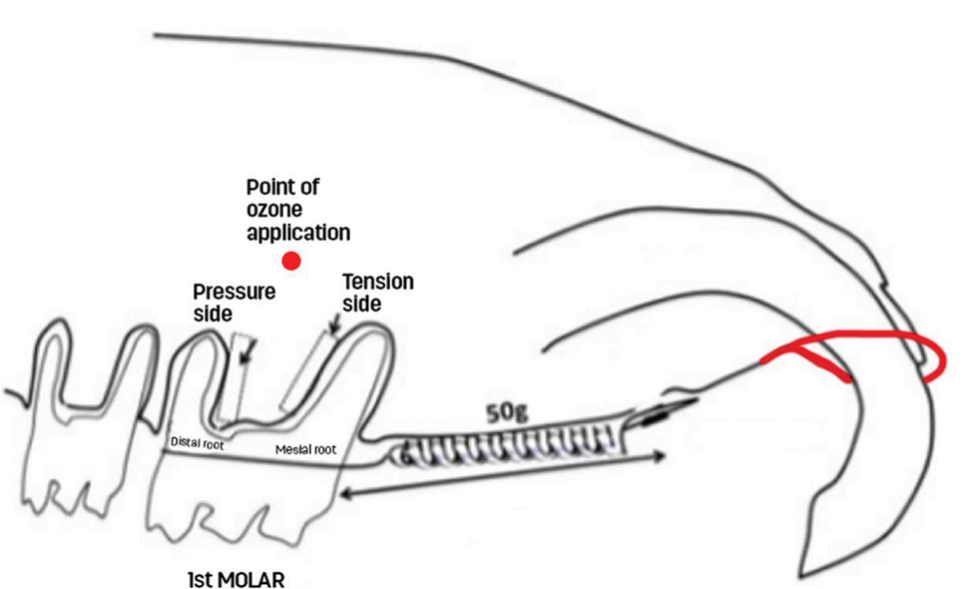
Schematic representation of the device used for tooth movement, point of ozone application, and areas of evaluation of the histological sections.

The mesial surface of the distobuccal root was evaluated as the pressure side, and the distal surface of the mesiobuccal root, as the tension side ( Fig. 2 ). The analyzed area extended from the third of the root close to the furcation region to the beginning of the apical third longitudinally and from the root surface to the alveolar bone transversely.

The evaluations considered the pattern of bone resorption and neoformation on the pressure and tension sides, respectively. The number of blood vessels, osteoclasts, and osteoblasts, the points of root resorption, the number of polymorphonuclear and mononuclear inflammatory cells, and the presence of osteoid tissue (tension) and hyaline areas (pressure) were evaluated. Osteoclasts and osteoblasts cells were differentiated based on cell morphology, considering the line of active osteoblasts next to newly formed osteoid. The microscopic analyses were performed in an optical microscope (Trinocular Biological Microscope LED Nikon Eclipse E200 Nikon, Tokyo, Japan) by a blinded and calibrated evaluator.

### Error Study

To evaluate the intraexaminer error, 30% of the sample was randomly selected and reevaluated by the same examiner after 1-month interval. Kappa tests and intraclass correlation coefficient (ICC) were used, and both showed a good to excellent degree of reliability for all variables analyzed.

### Statistical Analysis

The statistical analysis included qualitative and quantitative assessment of the parameters evaluated.

The number of osteoclasts, blood vessels, resorption areas, osteoblasts, polymorphonuclear and mononuclear cells on the pressure and tension sides were compared among the two groups that received the two ozone concentrations and the control group at 3 and 5 days with Kruskal–Wallis nonparametric and Dunn tests.

The comparison of the presence or absence of hyaline areas on the pressure side and osteoid tissue on the tension side among the two ozone concentrations and the control groups at 3 and 5 days was performed by Chi-square tests.

Statistical analysis was performed with Statistica software (Statistica for Windows, version 10.0, Statsoft, Tulsa, United States) and results were considered significant at *p* <0.05.

## Results

### Pressure Side

**Fig. 3:**
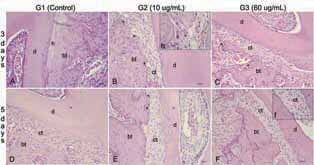
Histological features pressure side: ( **A** ) Presence of hyaline area ( *h* ). ( **B** ) Loose cellularized connective tissue (ct) with ovoid or stellate young fibroblasts and absence of hyaline area (Hematoxylin & Eosin, x10); (b) osteoclasts (*) are highlighted (Hematoxylin & Eosin, x40). ( **C** ) Presence of dense connective tissue (ct) with spindle-shaped mature fibroblasts and absence of hyaline area. ( **D** ) Cellularized connective tissue (ct) with ovoid fibroblasts. ( **E** ) Loose cellularized connective tissue (ct) with ovoid fibroblasts and osteoclasts (*). ( **F** ) Loose connective tissue (ct) with ovoid and stellate young fibroblasts (Hematoxylin & Eosin, x10); (f) Ovoid and young stellate fibroblasts (Hematoxylin & Eosin, x40). Dentin (d), bone tissue (bt).

At 3 days, group 2 (ozonated gas 10 µg/mL) showed a significantly higher number of blood vessels and polymorphonuclear inflammatory cells than the other groups. Both, groups 2 and 3 (ozonated gas 10 and 60 µg/mL), presented a significantly higher number of active osteoclasts than the control group ( Table 1 ). Besides, group 1 showed predominance of hyaline areas and disorganized discrete loose connective tissue, unlike the groups 2 and 3. Group 2 revealed predominance of loose cellularized connective tissue with ovoid or stellate young fibroblasts and absence of hyaline areas. At group 3, the dense connective tissue was more organized with spindle-shaped mature fibroblasts and absence of hyaline areas ( Fig. 3 ).

**Table 1:** Intergroup comparisons of the variables on the pressure and tension sides, at 3 and 5 days (Kruskal–Wallis and Dunn tests)

Variables	G1 (Control)					G2 (10 µg/mL)						G3 (60 µg/mL)					*p* -Value
	Mean (Median)			S.D. (i.r.)		Mean (Median)			S.D. (i.r.)			Mean (Median)			S.D. (i.r.)		
Pressure side—3 d																	
Osteoclasts	2.0 (1.5) A			1.4 (2.0)		6.3 (6.5) B			1.0 (1.5)			6.5 (5.5) B			3.3 (5.0)		**0.033** ^a^
Blood vessels	8.0 (8.5) A			2.9 (4.0)		20.3 (19.0) B			4.0 (5.5)			6.8 (7.0) A			0.5 (0.5)		**0.017** ^a^
Resorption	0.2 (0.0)			0.5 (0.5)		1.0 (0.5)			1.4 (2.0)			1.3 (1.5)			1.0 (1.5)		0.328
Osteoblasts	0.0 (0.0)			0.0 (0.0)		2.8 (2.5)			3.2 (5.5)			3.5 (4.5)			2.4 (3.0)		0.157
Polymorphonuclear cells	0.2 (0.0) A			0.5 (0.5)		18.5 (21.5) B			9.7 (14.0)			0.0 (0.0) A			0.0 (0.0)		**0.009** ^a^
Mononuclear cells	0.0 (0.0)			0.0 (0.0)		6.5 (7.0)			5.2 (8.0)			3.5 (4.0)			3.0 (5.0)		0.081
Tension side—3 d																	
Osteoclasts	1.2 (1.0)			1.2 (1.5)		2.3 (2.0)		1.3 (1.5)				3.3 (3.5)			1.7 (2.5)		0.192
Blood vessels	21.2 (23.5) A			8.6 (11.5)		32.3 (33.5) B		5.9 (7.5)				14.5 (14.5) A			3.7 (5.0)		**0.036** ^a^
Resorption	2.0 (2.0)			1.8 (3.0)		1.3 (1.0)		0.5 (0.5)				2.5 (2.5)			2.4 (4.0)		0.829
Osteoblasts	10.0 (13.0) A			6.7 (8.0)		23.5 (21.0) B		8.5 (13.0)				9.5 (10.0) A			5.0 (7.0)		**0.023** ^a^
Polymorphonuclear cells	1.2 (0.0)			2.5 (2.5)		4.8 (5.0)		1.5 (2.5)				1.8 (1.5)			1.7 (2.5)		0.086
Mononuclear cells	0.0 (0.0) A			0.0 (0.0)		71.5 (83.0) B		39.0 (56.0)				7.8 (5.5) A			4.9 (5.5)		**0.006** ^a^
Pressure side—5 d																	
Osteoclasts	2.3 (2.5) A			1.0 (1.5)		5.5 (5.5) B		1.3 (2.0)			3.3 (3.0) A			1.3 (1.5)			**0.029** ^a^
Blood vessels	6.5 (6.0) A			1.9 (3.0)		14.5 (14.5) B		1.3 (2.0)			19.0 (18.5) B			2.2 (3.0)			**0.007** ^a^
Resorption	0.3 (0.0) A			0.5 (0.5)		2.8 (2.5) B		1.0 (1.5)			2.0 (2.0) B			0.0 (0.0)			**0.009** ^a^
Osteoblasts	1.5 (0.0) A			3.0 (3.0)		10.3 (9.5) B		3.4 (4.5)			10.0 (9.5) B			3.9 (6.0)			**0.028** ^a^
Polymorphonuclear cells	2.8 (2.5) A			3.2 (5.5)		5.0 (5.0) A		2.4 (3.0)			0.0 (0.0) B			0.0 (0.0)			**0.049** ^a^
Mononuclear cells	3.3 (3.5)			2.8 (4.5)		7.0 (7.0)		1.2 (2.0)			3.8 (4.0)			3.0 (4.5)			0.078
Tension side—5 d																	
Osteoclasts		2.3 (1.0)	3.2 (3.5)		1.8 (2.0)		0.5 (0.5)			1.8 (1.5)			2.1 (3.5)			0.868	
Blood vessels		28.5 (28.0) A	5.7 (7.0)		25.8 (25.5) A		1.7 (2.5)			8.5 (8.5) B			0.6 (1.0)			**0.019** ^a^	
Resorption		2.0 (1.5)	2.2 (3.0)		2.3 (2.0)		0.5 (0.5)			1.0 (1.0)			1.2 (2.0)			0.308	
Osteoblasts		14.0 (13.5)	5.8 (10.0)		14.0 (15.5)		10.2 (16.0)			17.8 (18.5)			9.0 (11.5)			0.860	
Polymorphonuclear cells		10.5 (7.5) A	9.3 (12.0)		0.0 (0.0) B		0.0 (0.0)			0.3 (0.0) B			0.5 (0.5)			**0.009** ^a^	
Mononuclear cells		18.3 (18.0) A	1.5 (2.5)		3.5 (3.0) B		3.3 (4.0)			0.0 (0.0) B			0.0 (0.0)			**0.008** ^a^	
^a^ Statistically significant at *p* < 0.05.																	

At 5 days, group 2 (ozonated gas 10 µg/mL) showed significantly higher number of blood vessels, polymorphonuclear cells, and osteoclasts than the control group. However, significantly more tooth resorption points were also noted in group 2 compared with the control, and this was not observed at 3 days of tooth movement ( Table 1 ). In the pressure areas of group 1, the collagen fibers were dense with ovoid and sometimes fusiform fibroblasts, with absence of hyaline areas. At group 2, there was disorganized loose connective tissue, well cellularized, marked by immature ovoid fibroblasts and osteoclasts. Like group 2, group 3 showed loose connective tissue with ovoid and stellate fibroblasts ( Fig. 3 ). No ozone therapy sample showed formation of a hyaline area ( Table 2 ).

**Table 2:** Intergroup comparison of the presence of hyaline areas on the pressure side and osteoid tissue on the tension side (Chi-square test)

3 and 5 d			
Hyaline areas—pressure side			
Group/Hyaline areas	Yes	No	Total
Control	4	0	4
10	0	4	4
60	0	4	4
*X*^2^ = 12.00 DF = 2 *p* = 0.002 ^a^			
3 d			
**Osteoid tissue—tension side**			
**Group/osteoid tissue**	**Yes**	**No**	**Total**
Control	1	3	4
10	4	0	4
60	4	0	4
*X*^2^ = 8.00 DF = 2 *p* = 0.018 ^a^			
**5 d**			
**Osteoid tissue—tension side**			
**Group/osteoid tissue**	**Yes**	**No**	**Total**
Control	4	0	4
10	4	0	4
60	4	0	4
^a^ Statistically significant at *p* < 0.05.			

### Tension Side

**Fig. 4:**
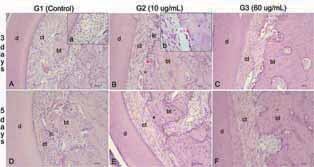
Histological features tension side: ( **A** ) Loose cellularized connective tissue (ct) with ovoid or stellate young fibroblasts (Hematoxylin & Eosin, x10); (a) Ovoid and young stellate fibroblasts (Hematoxylin & Eosin, x40). ( **B** ) Organized dense connective tissue (ct) with spindle-shaped fibroblasts and focal inflammatory infiltrate (ic). Neoformed osteoid tissue ( *black* *) and active osteoblasts are highlighted ( *red* *) (Hematoxylin & Eosin, x10); (b) active osteoblasts next to newly formed osteoid ( *red* *; Hematoxylin & Eosin, x40). ( **C** ) Dense connective tissue (ct) with more organized fibers and spindle-shaped fibroblasts. ( **D** ) Connective tissue with areas of organized collagen fibers and spindle-shaped fibroblasts, sometimes disorganized areas with inflammatory cells (ic). ( **E** ) Dense connective tissue with well-organized collagen fibers and spindle-shaped fibroblasts, and without inflammatory infiltrate. ( **F** ) Dense connective tissue with more organized fibers and spindle-shaped fibroblasts following the fiber disposition. Dentin (d), bone tissue (bt). (Hematoxylin & Eosin, x10).

At 3 days, the control group presented disorganized loose cellularized connective tissue with immature stellate fibroblasts but with a discrete area of osteoid tissue. All samples treated with ozone therapy presented osteoid tissue formation at 3 days, unlike the control group, where only one sample showed osteoid tissue formation ( Table 2 ). Group 2 (ozonated gas 10 µg/mL) showed significantly more blood vessels, mononuclear cells, and osteoblasts than control and group 3 ( Table 1 ). Consequently, in this group (2), osteoid tissue areas were observed, indicating the beginning of bone neoformation, with the predominance of dense connective tissue and spindle-shaped fibroblasts. At group 3, there was organized dense connective tissue and spindle-shaped fibroblasts ( Fig. 4 ).

However, at 5 days, both groups treated with ozone therapy had significantly fewer polymorphonuclear and mononuclear cells than the control. Group 3 showed lesser blood vessels than other groups ( Table 1 ). Group 1 presented dense collagen fibers with spindle-shaped fibroblasts, however, still showing the presence of discrete area of inflammation. On the other hand, ozone groups presented connective tissue with dense collagen fibers and spindle-shaped fibroblasts following the fiber disposition, marked by the absence of inflammatory areas ( Fig. 4 ).

## Discussion

### Methods

Ozone is considered a modifier of the biological response 28 and it can act in several ways in the body. The present study aims to evaluate only the biological response of applying two ozone concentrations when stimulating orthodontic force, not the mechanism of action.

The concentrations of 10 μg/mL and 60 μg/mL were chosen to evaluate the effect of ozone therapy on orthodontic force induction, considering the dose per point of application, which can cause biostimulation, anti-inflammatory or immunosuppressive effects. 9 For biostimulation, the concentration of 10 µg/mL 23 was chosen instead of 25 µg/mL 14 because the first presented an increase in interleukins, and this is important for orthodontic movement. The 25 µg/mL increased the bone healing process. 14 The dose of 60 µg/mL was chosen to observe the anti-inflammatory effect 9 on orthodontic force induction since there is no previous study on the subject.

The dose in ozone therapy is calculated by multiplying the values of volume and concentration (Dose = Volume × Concentration) and is expressed in μg. In this study, the volume of 1 mL of ozonated gas was used, 14 allowing to test two concentrations indicated to promote different effects: biostimulator (up to 5 µg/mL) and anti-inflammatory (between 50 and 150 µg/mL). 9 The volume of 1 mL was useful to the easy obtention of the final concentration and also to standardize for future research.

The allometric extrapolation index was not used in this study, as ozone is not absorbed, but undergoes an immediate redox reaction. This index takes into account the basal metabolic rate of each organism, which, in the case of rats, is around 3× faster than in humans, and therefore, the doses of medication used are usually higher. 29

The subcutaneous application of ozonated gas was chosen because it is a technique of easy application in dental offices for orthodontic purposes, although there are many forms of application of ozone therapy. 28 It can be thought that this type of application is difficult to be accepted by orthodontic patients, however, comparing it with other existing techniques on the market, we observed that in addition to the very low cost, the procedure is less invasive than microperforations or corticotomy surgeries or piezo incision surgeries.

The therapeutic effects of ozone cannot be restricted to a limited area. However, this effect has a tendency to acidic means, or means where inflammation exists. 30 For this reason, we use only one of the rat’s molars, and we do not use the equivalent molar as a control.

The follow-ups of 3 and 5 days were chosen to report changes in the first days of induced inflammation, and previous studies demonstrated that 5 days is the period with the greatest cell activity. 31 32 Moreover, the action of ozone occurs immediately after its injections.

The evaluation was performed after 3 and 5 days from the beginning of induced inflammation since the third day reflects the time of greatest cell recruitment with the use of ozone therapy, 33 and is usually a latency period of orthodontically-induced tooth movement. At 5 days, it was important to evaluate the activity of the subsequent effects of ozone therapy after its total reaction. Besides, we intended to evaluate the effects of local application of ozone in the first days of orthodontic force induction.

The 50 g force was used to promote tooth movement and also root resorption in rats. 34 The choice of this force allowed the action of ozone to be tested against forces that also generate root resorption, as it is speculated that ozone therapy could stimulate protective cells of the periodontal ligament (cementoblasts) to defend the tissue against aggression.

## Results

For tooth movement to occur, osteoclasts must promote bone resorption along the pressure side of the periodontal ligament, while osteoblasts must promote bone neoformation along the tension side and in the remodeling resorption areas on the pressure sides. 35 Theoretically, a large number of inflammatory cells in the region associated with large number of osteoclasts and osteoblasts are likely to accelerate the bone remodeling process. These results were presented by the group of 10 µg/mL of ozone therapy, which presented a greater number of inflammatory cells at 3 and 5 days, and better efficient tissue reorganization.

It can be seen that, in the groups where ozone therapy was performed, there was no formation of hyaline areas of necrosis, which can be explained by the greater formation of blood vessels and the consequent increase in the supply of oxygen. 36 This result is favorable for orthodontic movement. Normally, the situation of hypoxia or anoxia, due to compression of the periodontal ligament, leads to the death of cells and the formation of hyaline areas. These hyaline areas are undesirable, as they delay the induced tooth movement and the phagocytosis of these areas is necessary for dental movement. 37

The greater number of cells observed in the ozone therapy groups (polymorphonuclear, mononuclear cells, osteoclasts, and osteoblasts) can be justified by the fact that ozone activates angiogenesis, 38 allowing these cells to easily access the inflammatory area. Another important fact mentioned in the literature that may justify the greater number of cells is that ozone improves the performance of red blood cells that transport oxygen through tissues and activates a series of biological mechanisms that lead to normalization of oxygen delivery. 39 Ozone also increases the release of nitric oxide, 40 which improves vasodilation in ischemic areas, 41 justifying the increase in vascularization in the regions where ozone was used. For these reasons, it is supposed that the intense force that would generate an initial necrotic response did not affect the groups that received ozone therapy.

The results obtained for group 2 (10 μg/mL) were compatible with previous studies showing stimulation of cell proliferation 33 in bone remodeling therapies in lumbar hernias, 23 26 increased vascularization of the treated region, 14 and polymorphonuclear cells indicating an increase in the inflammatory infiltrate. This low ozone concentration, considered as biostimulator, can be useful during orthodontic movement in the process of bone remodeling. Chemical messengers such as cytokines, NO, and prostaglandin E have the property of stimulating both osteoclastic and osteoblastic responses. 35 These messengers are stimulated by ozone therapy. 23 42 43

In group 3 (60 µg/mL), the ozone application probably had an anti-inflammatory effect since, at 3 days, the inflammation was minimum or inexistent, accompanied by the organization of the periodontal ligament that already had dense connective tissue, with fusiform collagen fibers at 5 days. For heavy forces without ozone therapy, cell reorganization begins at 9 days. 37 Perhaps this ozone concentration that promotes inflammatory modulating is interesting to be used to stabilize orthodontic treatment, as the periodontal ligament reorganizes more quickly.

Root resorption increased in ozone groups. Unlike the idea that root resorption could be attributed to eliminating hyaline areas, 44 an increase in resorption points was observed in this study even in the ozone groups that did not present hyaline areas. Studies show that the increase in bone turnover and osteoclastic activity in the alveolar bone cause an increase in the severity of root resorption, 44 45 which was seen in the present study.

The effect of ozone therapy using lighter orthodontic forces, which do not induce root resorption, should be evaluated to assess if the ozone therapy in low concentration and with ideal forces will cause root resorption or not.

The results of ozone therapy against an induced inflammatory stimulus were quite interesting. The greater number of cells during the evaluated period provided a positive histological result for orthodontically-induced tooth movement.

As orthodontics is a science that works with bone remodeling (bone resorption and neoformation), it was observed that, when low concentration ozone therapy was used, this process was stimulated by the greater number of osteoclasts on the pressure side and the greater number of osteoblasts on the tension side. Also, the absence of hyaline areas in the ozone groups is interesting, as these areas with absence of cells delay tooth movement. The increase in local microcirculation may have provided all these positive parameters for orthodontic movement.

The undesired increase in root resorption is probably explained by the increased inflammation in the region. It is known that the ideal force for orthodontic movement is light and constant force. 35 Therefore, new research should be performed with lighter forces, in animal models, so that the real benefit of ozone therapy in orthodontic movement can be evaluated. For this, suggested low ozone concentration is (below 30 µg/mL) which acts as biostimulator and longer observation time of orthodontically-induced tooth movement.

## Conclusion

Ozone therapy increased the number of osteoclasts on the pressure side and osteoblasts on the tension side, at the concentration of 10 µg/mL, during the evaluated period, demonstrating histological parameters favorable to bone remodeling. At the concentration of 60 µg/mL, ozone therapy accelerated the periodontal ligament reorganization process.
